# Ethical considerations in the allocation of critical care resources when capacity is overwhelmed

**DOI:** 10.1017/cem.2020.354

**Published:** 2020-03-30

**Authors:** Merril A. Pauls, David Migneault, Francis Bakewell

**Affiliations:** * Health Sciences Centre, Max Rady College of Medicine, Department of Emergency Medicine, University of Manitoba, Winnipeg, MB; CAEP Bioethics Committee; † Vancouver General Hospital, BC Children's Hospital, UBC Urgent Care Centre, Vancouver Coastal Health, UBC Department of Emergency Medicine, University of British Columbia, Vancouver, BC; CAEP Bioethics Committee; ‡ Medicine, Ethics, and Humanities Program, The Ottawa Hospital; Faculty of Medicine, University of Ottawa, Ottawa, ON; § CAEP Bioethics Committee

**Keywords:** Critical care, ethics, pandemic, resource allocation

## INTRODUCTION

Canadian emergency physicians fear we will find ourselves in a situation similar to Northern Italy, where critical care resources have been overwhelmed in the face of critically ill COVID-19-infected patients. This raises important ethical issues about how we decide who gets access to critical care when we cannot provide it to everyone.

During a pandemic, we need to respect individual rights and freedoms while considering the needs of the broader public. Any decision-making frameworks we use in these exceptional times should consider the traditional principles of medical ethics, but also must reflect the core principles of public health ethics: respect; the harm principle; fairness; consistency; least coercive and restrictive means; working together; reciprocity; proportionality; flexibility; and procedural justice.

## TRIAGE OF CRITICAL CARE RESOURCES WHEN DEMAND OVERWHELMS AVAILABILITY

There are strong ethical justifications for the development and application of robust triage systems in the event that a pandemic overwhelms our ability to provide critical care to all patients. Emergency physicians are experts at identifying those who most require their care and focussing resources on this population. During a pandemic, when resources are overwhelmed, the additional and most difficult aspect of triage would be that some patients who may benefit from critical care, or who would have been offered this care under different circumstances, would now be denied this care. This raises important considerations, as follows:

### 1. Before applying any criteria, we would have to demonstrate that capacity is truly overwhelmed.

Has the system done its best to mobilize additional resources, and use all available resources in creative ways, to benefit as many patients as possible?

### 2. Is the “formula” we use to decide who gets care (and who doesn't) evidence-based and defensible?

We know with COVID-19 infection that advanced age predicts a poorer outcome, but other important factors play a role. There are data from the Chinese experience, which shows that we may be able to identify specific patients who will not do well (i.e., those who develop fulminant myocarditis),^1^ and there are other predictors of higher mortality (including presence of cardiovascular disease and a high Sequential Organ Failure Assessment [SOFA] score)^2^ beyond just age.

Criteria such as age, stage of life, mental ability, physical ability, and/or disability should not be used in isolation as allocation criteria. The moral worth, value, and dignity of all persons are equal regardless of these criteria. However, these criteria may be considered within the decision-making process when other objective clinical features, such as associated comorbidities, are likely to impact an individual's ability to survive an acute illness.

Additional considerations include an ongoing reassessment process to ensure that patients who receive critical care are continuing to benefit from it and the ongoing incorporation of new evidence in triage criteria.

### 3. How do we apply the triage criteria fairly?

Some have suggested that we should take these treatment decisions out of the hands of front-line clinicians, using designated triage physicians or triage teams. This would promote the fair application of rules and insulate front-line providers from the moral distress associated with denying potentially beneficial care to their own patients.^3^ Others believe this is not practical, and call for clear, simple criteria that can be readily understood and easily applied. An awareness of personal biases and other factors that could lead to discrimination/favoritism is crucial. There should be monitoring to determine whether certain groups are being systematically disadvantaged or advantaged by the criteria and their application.^4^

### 4. How should we communicate with patients and their families if they are deemed to be ineligible for critical care?

There are scripts available to help foster these conversations and promote honesty and transparency.^5^ Affected patients and families should be made aware that resources are being withheld in the context of the COVID-19 pandemic, that these actions are based on objective and broadly accepted guidelines, and that any other available beneficial care will still be provided.

### 5. Should there be an appeal process for these decisions?

While there are arguments for and against a formal appeal process in these situations,^6^ physicians should remember that patients/families can always challenge these decisions, including initiating complaints to the healthcare institution, the regulatory authority, or even initiating a legal action. Physicians need to ensure that their decisions are defensible in the event that these types of complaints occur.

### 6. Is there institutional/professional/legal support for these difficult triage decisions?

Provided that an agreed upon, evidence-based, and transparent set of rules is used to decide who gets critical care and who does not, the healthcare institutions we work in need to take a clear stand supporting front-line providers making these decisions. There also needs to be substantial agreement and support between emergency medicine and critical care so that patients and families receive consistent messages and emergency physicians do not initiate care that cannot or will not be continued.

## HOW DO WE DECIDE WHO GETS CARE AND WHO DOESN'T?

It is estimated that 50% of Canadians have written advance directives. There are significant barriers to these wishes being known and acted upon by healthcare providers. In the event that critical care capacity is overwhelmed, it will be crucial that we do not use scarce resources for patients who do not want them, or who would not want them if adequately informed about their prognosis. We must identify those who already have advance directives (particularly those who request no intubation/ventilation in the event of critical illness) and clearly transmit that information to front-line providers who will care for them. We should engage in compassionate and clear conversations with patients who are at risk for poor outcomes with COVID-19 infection as far in advance as possible, to identify those who would not want to be considered for critical care interventions.

Most hospitals, health regions, and provinces have engaged in emergency measures and pandemic planning exercises in response to prior outbreaks. Some are also currently working on specific critical care triage policies in the event that local capacity is overwhelmed. It is vital that emergency physicians have input into these policies. Emergency providers need to ensure that these policies consider the issues identified above, and that there is clear guidance regarding key triage criteria, and how they are to be used in the emergency setting. These policies need to be widely disseminated, discussed, and supported within the relevant region. While some have expressed concern that a broader discussion of these policies could cause fear or anxiety, a lack of community engagement and stakeholder involvement will undermine credibility and make triage conversations with our patients and their families even more difficult.

The most relevant published guidelines are from the Ontario Health Plan for an Influenza Pandemic (OH-PIP) subgroup report published almost 14 years ago.^7^ They offer a specific approach to triaging critically ill patients and clinical criteria that can be used to determine whether patients should be excluded from or considered for intensive care unit (ICU) treatment when capacity is exceeded. Subsequent work has shown these guidelines maximize benefits but may need additional ethical considerations to enhance equity and fairness.^8^

International commentaries and triage guidelines have been released in response to the current crisis.^9,10^ While Canadian physicians can learn important lessons from these, they should be aware that differences in healthcare systems, healthcare cultures, and national values may limit or alter the applicability of these guidelines.

## BOTTOM LINE

1. We must identify patients who do not want critical care interventions and clearly transmit these wishes to the providers who care for them. We need to initiate conversations with people at risk for poor outcomes with COVID-19 infection, to determine their treatment wishes.
2. There are guidelines that provide explicit guidance for physicians regarding allocation decisions. These guidelines can maximize benefits but require additional considerations to promote equity and fairness.
3. Emergency physicians should determine whether there are local relevant policies and guidelines available, and use other local ethics resources if possible. Any triage guidelines must include emergency medicine input and address how any criteria would be used in the emergency setting.
4. The local community and other relevant stakeholders should be engaged in the process of guideline development and dissemination. Just as important as what is decided is how it is decided and who has been involved.
5. The application of triage guidelines should be done in an honest, transparent, and compassionate manner. Physicians will need significant support to apply guidelines fairly, and there must be substantial agreement between ICU and emergency physicians.
